# Preparation and Experimental Evaluation of Phase-Change Characteristics in Carbon-Based Suspensions

**DOI:** 10.3390/ma11081315

**Published:** 2018-07-30

**Authors:** Tun-Ping Teng, Ting-Chiang Hsiao, Chun-Chi Chung

**Affiliations:** 1 Undergraduate Program of Vehicle and Energy Engineering, National Taiwan Normal University, No. 162, Sec. 1, He-ping E. Road, Da-an District, Taipei City 10610, Taiwan; 2 Department of Industrial Education, National Taiwan Normal University, No. 162, Sec. 1, He-ping E. Road, Da-an District, Taipei City 10610, Taiwan; mall456kimo@hotmail.com (T.-C.H.); sekee0118@gmail.com (C.-C.C.)

**Keywords:** micro/nanocarbon-based materials (MNCBMs), carbon-based suspensions (CBSs), differential scanning calorimeter (DSC), high-pressure combustion method (HPCM), subcooling degree (*SD*)

## Abstract

In this study, micro/nanocarbon-based materials (MNCBMs) were prepared using the high-pressure combustion method (HPCM) with an isoperibol oxygen bomb calorimeter at different oxygen pressures (0.5–3.0 MPa). The prepared MNCBMs were added to water to form carbon-based suspensions (CBSs); sodium dodecyl benzene sulfonate (SDBS) and defoamer were added to the CBSs to enhance their stability. The thermal conductivity, viscosity, density, and contact angle of the CBSs were measured using appropriate instruments to determine their fundamental characteristics. The phase-change characteristics of the CBSs were measured and analyzed using a differential scanning calorimeter (DSC) to evaluate the feasibility of employing them as phase-change materials in ice-storage air-conditioning systems. The results revealed that the maximal change ratios of thermal conductivity, viscosity, density, and contact angle of the samples were −3.15%, 6.25%, 0.23%, and −57.03%, respectively, as compared with the water. The CBS of S5 (oxygen pressure of 2.0 MPa) had the lowest melting temperature and subcooling degree (*SD*) and the highest freezing temperature in the experiments conducted using the DSC; thus, S5 was determined to be the most suitable CBS for use as a phase-change material of cold energy storage in this study.

## 1. Introduction

Latent heat storage (LHS) is the most common thermal energy storage method. For LHS, the most appropriate phase-change material (PCM) for thermal energy storage can be selected based on the temperature requirement, and LHS features high-energy storage density and efficiency [1]. Water is the most commonly used PCM in ice-storage air-conditioning systems (ISACSs). During “charging mode”, the operating temperature of ISACS chillers is set below water’s freezing point, and ice is formed in the storage tank. When the ISACS operates in “air-conditioning mode (discharging mode)”, the coolant is circulated around the storage tank to provide the air-conditioning load. The ISACS runs the chiller in charging and discharging modes during periods of off-peak and on-peak electricity demand, respectively. This operation process improves the overall efficiency of the power system by effectively scheduling electricity demand during off-peak and on-peak hours [2,3,4].

Chiller operating efficiency decreases with decreasing evaporation temperature; therefore, the efficiency of the ISACS in charging mode is lower than that of conventional air-conditioning systems operating in air-conditioning mode [2]. In practice, water does not freeze at 0 °C, and the cooling temperature of the coolant must be below the nucleation temperature (*T_n_*) to enable the formation of ice crystals from water, which freezes through ice crystal growth. Therefore, water must reach *T_n_* and subsequently return to the ice–water coexistence zone at approximately 0 °C for freezing. *T_n_* is lower than the ice–water coexistence temperature (solidification temperature *T_s_*); the temperature difference between *T_n_* and *T_s_* (or melting temperature *T_m_*) is called the subcooling degree *(SD)*. In general, a lower *SD* benefits the charging process because it enables the evaporation temperature of the chiller to be higher and increases operating efficiency [1,3].

Ice nucleation can be divided into homogeneous nucleation and heterogeneous nucleation. Nucleation that occurs in the main phase (of water) is called homogeneous nucleation. By contrast, when nucleation occurs in containers and cooling coils, or in impurities in water, it is called heterogeneous nucleation [5,6]. The free energy variation required for heterogeneous nucleation is lower than that required for homogeneous nucleation; thus, ice nucleation can occur with less energy variation [7,8,9]. In general, ISACSs employ heterogeneous nucleation; therefore, their *SD* is generally low. Many related studies have added nondissolvable solid particles or crystalline materials to water as nucleating agents to promote heterogeneous nucleation and reduce the *SD*. In recent years, with the development of nanotechnology, nanoparticles have been stably suspended in various PCMs as nucleating agents [3,5,6,7,8,9,10,11,12,13,14,15,16,17,18]. Copper [5], aluminum oxide [10,11], titanium dioxide [12,13,14], silicon dioxide [14], nanocarbon or carbon nanotubes [15,16,17,18], graphene [14,15], and graphene oxide [3] have all been added to PCMs to lower their *SD*, thereby increasing energy storage efficiency. Nanomaterial addition has had little effect on the kinematic viscosity of PCMs but has improved their thermal conductivity and charging and discharging rates [16,18,19]. 

In recent years, many studies of carbon-based nanomaterials, such as nanocarbons, carbon nanotubes, graphene, and graphene oxide have been conducted, which indicate these materials have characteristics such as antiaging, special mechanical properties, high thermal conductivity [20,21,22,23], excellent heat-transfer performance [22,24,25], and low *SD* and freezing duration in the phase-change process [3,15,17,18]. Therefore, the use of carbon-based nanomaterials to make nanofluids and thermal storage applications is a worthwhile research direction. In this study, micro/nanocarbon-based materials (MNCBMs) were prepared using the high-pressure combustion method (HPCM) at different oxygen pressures (0.5–3.0 MPa). The HPCM used for the preparation of MNCBMs in this study has the advantages of simplicity, rapidity, and safety because of the use of an existing and well-developed instrument—the isoperibol oxygen bomb calorimeter. The MNCBMs were added to water to form carbon-based suspensions (CBSs); sodium dodecyl benzene sulfonate (SDBS) and defoamer (DF) were added to the CBSs to enhance their stability. The morphology and fundamental characteristics of the MNCBMs and CBSs with various manufacturing parameters were ascertained. Lastly, the phase-change characteristics of the CBSs were measured and analyzed using a differential scanning calorimeter to evaluate the feasibility of employing them as PCMs in future ISACSs.

## 2. Sample Preparation

**Figure 1:**
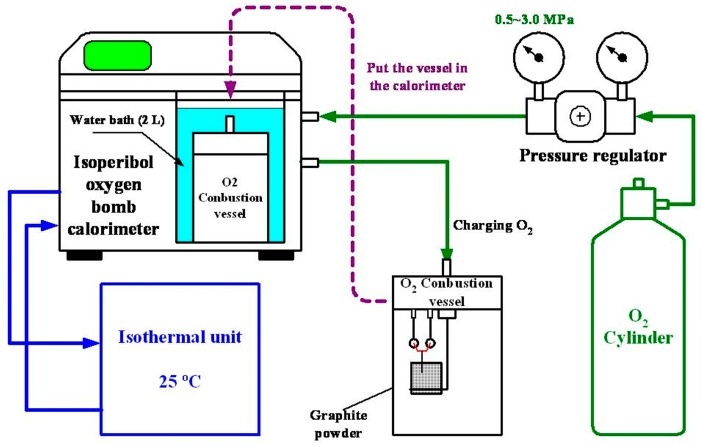
Installation of the high-pressure combustion method (HPCM) manufacturing system.

The HPCM manufacturing system of MNCBMs proposed in this study is shown in Figure 1. The main body of the system is an isoperibol oxygen bomb calorimeter (6200EA, PARR, Moline, IL, USA) with an external oxygen cylinder, oxygen pressure regulator, isothermal unit, and oxygen combustion vessel (oxygen bomb). Graphite powder (GP; average size = 3.2 μm) was exploded in the isoperibol oxygen bomb calorimeter by applying various oxygen pressures to the oxygen bomb, namely 0.5, 1.0, 1.5, 2.0, 2.5, and 3.0 MPa, all of which are below the recommended pressure (30 atm) for calorimetry. These oxygen pressure settings were chosen to avoid the complete combustion of the GP, which would result in an insufficient MNCBM production rate [26]. The MNCBMs had varying morphologies and were composed of various materials because of various oxygen pressures to produce different explosive energies in oxygen bomb. First, 1.0 g of GP was placed in the oxygen bomb’s sample pan, which was subsequently subjected to various oxygen pressure levels through a standard combustion calorie measurement procedure to produce MNCBMs. Lastly, the combustion residue was weighed using an electronic balance (GR200, A&D, Tokyo, Japan), and the weight ratio of the residual weight after combustion to the initial weight of the GP was defined as the production rate (*PR*). For the isoperibol oxygen bomb calorimeter, we only need to input the weight of the sample and the ignition thread, and then it automatically calculated and recorded the total combustion heat value (*H_cv_*) of the sample under each experimental parameter through a standard combustion calorie measurement procedure. Since the sample was burned in a fixed volume chamber, the total combustion heat released by burning the sample and the sample’s moisture was automatically calculated by measuring the temperature change of the water bath. Therefore, *H_cv_* belongs to the higher heating value. 

**Figure 2:**
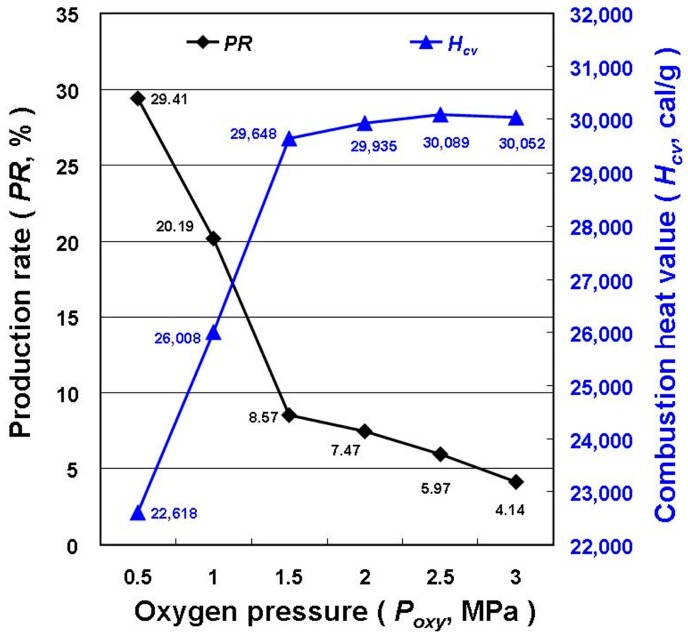
*PR* and combustion heat value (*H_cv_*) of the micro/nanocarbon-based materials (MNCBMs) for HPCM at various oxygen pressures.

Figure 2 displays the *PR* and *H_cv_* of the MNCBMs and the *H_cv_* of the GP for the HPCM at various oxygen pressures. These values were calculated as the average of five experimental results. Because the GP burned more completely under high oxygen pressure, the residual weight of the MNCBMs after combustion decreased and the *H_cv_* increased in a reasonable and predictable manner. However, from the calculated values, when the oxygen pressure reached 1.5 MPa, the decline rate of the *PR* and increase rate of *H_cv_* tended to be moderate, indicating that most of the GP had been burned. The *PR* of the MNCBMs produced at an oxygen pressure of 3.0 MPa was too low (less than 5%). Therefore, this process parameter (3.0 MPa) was excluded in the follow-up experiment based on the cost of preparation and feasibility for practical applications in the future.

**Figure 3:**
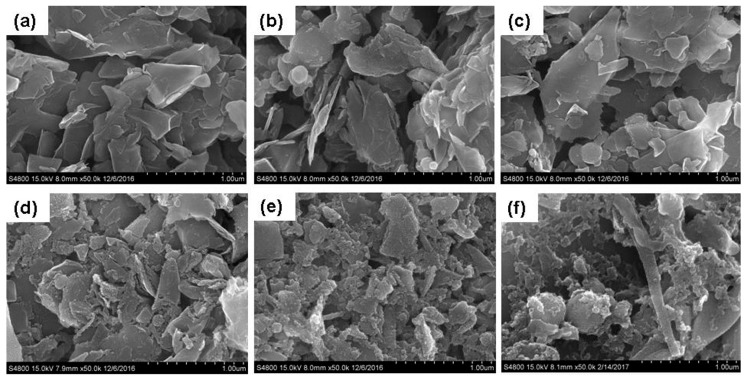
Field-emission scanning electron microscope (FESEM) image of the MNCBMs: (**a**) graphite powder (GP), (**b**) 0.5 MPa, (**c**) 1.0 MPa, (**d**) 1.5 MPa, (**e**) 2.0 MPa, and (**f**) 2.5 MPa.

**Figure 4:**
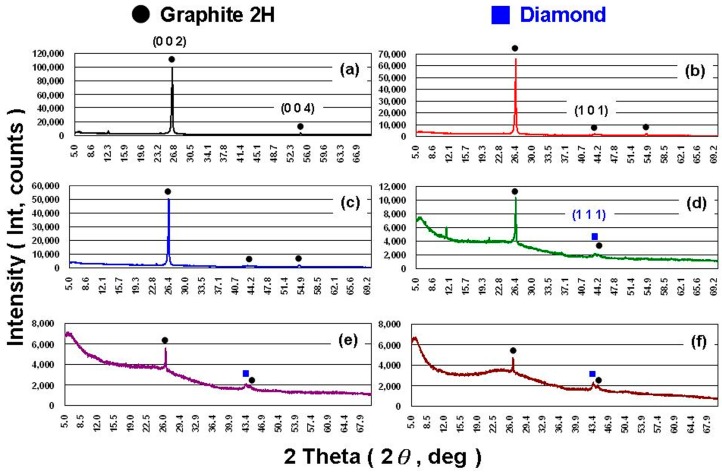
X-ray diffraction (XRD) patterns of the MNCBMs: (**a**) GP, (**b**) 0.5 MPa, (**c**) 1.0 MPa, (**d**) 1.5 MPa, (**e**) 2.0 MPa, and (**f**) 2.5 MPa.

Figure 3 shows a field-emission scanning electron microscope (FESEM; S-4800, Hitachi, Tokyo, Japan) image of the MNCBMs produced at various oxygen pressures. In terms of morphology, the MNCBMs produced at pressures of 0.5 and 1.0 MPa exhibited no obvious differences from the original GP (Figure 4a). However, generally, the MNCBMs were gradually crushed as the oxygen pressure increased. When the oxygen pressure exceeded 1.5 MPa, the MNCBMs produced through crushing were identifiable because high-pressure oxygen combustion in the oxygen bomb produced sufficiently high temperatures and pressure to break down the GP and reduce the particle size of the MNCBMs.

Figure 4 shows the X-ray diffraction (XRD; D8 Advanced, Bruker, Rheinstetten, Germany) patterns for the MNCBMs produced at various oxygen pressures. In terms of the XRD patterns, the MNCBMs crystallized at oxygen pressures under 0.5 and 1.0 MPa exhibited no obvious differences from the original GP (Figure 4a). The XRD patterns showed that the (0 0 2) diffraction peak was located at 2*θ* = 26.5°, indicating that the principal material of the MNCBMs was crystalline graphite (graphite 2H, PDF # 893439) [27]. A clear difference was observed between the materials of the MNCBMs and GP when the oxygen pressure was increased to 1.5 MPa or higher. The intensity of the (0 0 2) diffraction peak decreased as the oxygen pressure increased, and the intensity of the (1 1 1) diffraction peak increased as the oxygen pressure increased. The (1 1 1) diffraction peak was located at 2*θ* = 43.9°, indicating that the MNCBMs contained a diamond (PDF # 898499) [27]. The (1 1 1) diffraction peak was not sharp, and the diffraction peak intensity was not high; therefore, it was likely a diamond with poor crystallinity. In addition, overall, the diffraction patterns indicated that a part of the crystalline graphite had been converted to amorphous carbon when the oxygen pressure exceeded 1.5 MPa. Thus, the MNCBM produced when the oxygen pressure exceeded 1.5 MPa mostly comprised crystalline graphite (graphite 2H), amorphous graphite, and a small amount of defective diamond.

The MNCBMs prepared at various oxygen pressures were ground and dispersed in a fast ball-milling machine (MM400, Retsch, Haan, Germany), and wet milling (the weight ratio of water to MNCBMs was 10:1) was conducted for 20 min to further break the agglomerated MNCBMs. After the ball-milling procedure, each sample (slurry) was diluted with water to form 0.25 wt.% CBSs. The CBSs were stirred using a stirrer/hot plate (PC420D, Corning, Corning, CA, USA) operating at 450 rpm for 30 min, homogenized at 4500 rpm for 10 min in a homogenizer (YOM300D, Yotec, Hsinchu, Taiwan), bathed in an ultrasonic bath (5510R-DTH, Branson, MO, USA) for 20 min, and then subjected to intermittent oscillation for 10 min (25% amplitude, on/off duty was 10/30 s) in an ultrasonic liquid processor (Q700, Qsonica, Newton, CT, USA). The aforementioned dispersion method was repeated three times to prevent a temperature increase in the dispersion equipment and the CBSs, thereby achieving favorable dispersion and suspension performance for the CBSs to complete the initial manufacturing process [28].

**Figure 5:**
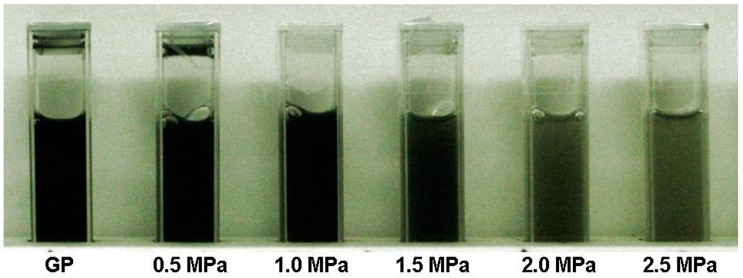
Photograph of the test samples.

Subsequently, 0.4 wt.% SDBS (Sigma-Aldrich, St. Louis, MO, USA) was added to each CBS to improve its stability. SDBS is commonly used to disperse nanocarbon-based materials in water to provide excellent stability [14,29,30,31,32]. The optimum addition concentration of the dispersant was determined by adding different concentrations of SDBS (0.05, 0.1, 0.2, 0.4, and 0.8 wt.%) to 0.25 wt.% CBS. A UV-VIS-NIR spectrometer (V670, Jasco, Tokyo, Japan) was used to measure the changes in absorbance for each CBS with various SDBS addition concentrations before and after standing for 48 h [29,30]. The results revealed that across the range of SDBS addition concentrations, the addition of 0.4 wt.% SDBS to 0.25 wt.% CBSs could achieve optimal suspension performance. Next, a DF (Antifoam B Silicone Emulsion, J. T. Baker, Center Valley, PA, USA) at concentration of 35% (0.14 wt.%) of the weight of the added SDBS was added to each CBS to reduce the volume of foam produced from SDBS to complete CBS preparation. The concentration of the added DF was effective in suppressing the foam produced by the 0.4 wt.% SDBS aqueous solution during stirring (stirrer was set at 400 rpm) [30]. The CBSs with the optimum concentration of SDBS (0.4 wt.%) had been observed for three weeks without any obvious settlement, indicating that the CBSs with the optimum concentration of SDBS had good stability. Figure 5 displays a photograph of 0.25 wt.% GP and MNCBMs in the base fluid (0.4 wt.% SDBS and 0.14 wt.% DF aqueous solution). The final experimental sample configuration used to evaluate the fundamental and phase-change characteristics of the samples is listed in Table 1.

## 3. Experimental Procedures 

The thermal conductivity (*k*), viscosity (*μ*), and density (*ρ*) of the samples were measured using a thermal properties analyzer (KD-2 Pro, Decagon Devices, Pullman, WA, USA; accuracy ±5.0%), resonant viscometer (VL700-T15, Hydramotion, Malton, UK; accuracy ±1.0%), and liquid density meter (DA-130N, KEM, Kyoto, Japan; accuracy ±0.001 g/mL), respectively, in an isothermal unit (HW401L, HILES, Taipei, Taiwan; accuracy ±0.5 °C) at 25 °C. The contact angle (*θ*) of the sample was estimated by measuring the contact angle of a droplet of the sample on the test substrate at room temperature and in ambient atmosphere by using a video tensiometer (FTA188, First Ten Ångstroms, Portsmouth, VA, USA) with an experimental deviation lower than 0.1°. A glass substrate with a flat polyimide film attached to its surface was adopted as the test substrate to provide greater hydrophobicity in order to improve the accuracy of the *θ* experiments.

**Figure 6:**
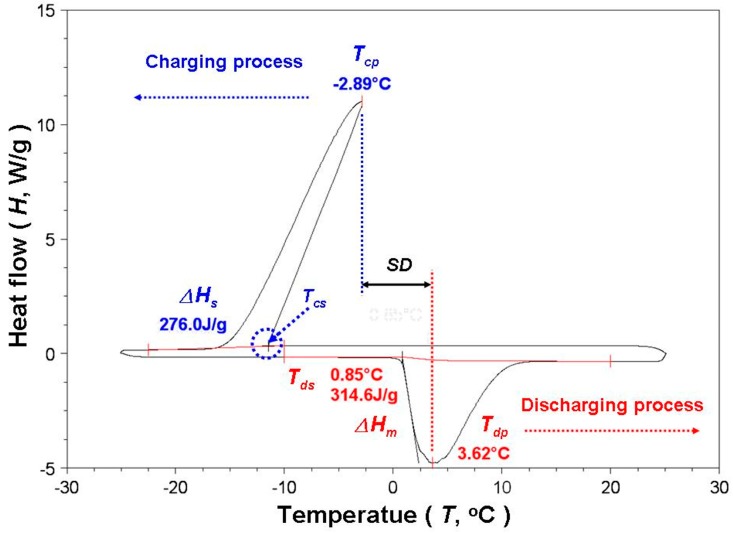
Charging and discharging curve of differential scanning calorimeter (DSC) for water.

A phase-change experiment was conducted using a DSC (DSC; Q20, TA, New Castle, DE, USA) with a mechanical refrigeration system (RCS40, TA, New Castle, DE, USA) in a high-purity nitrogen (5 N) atmosphere. The temperature and calorimetric accuracy of the DSC were ±0.1 °C and ±1.0%, respectively. The experimental temperature range was –25–25 °C, and the heating and cooling rates were both set at 5 °C/min. Figure 6 shows the charging and discharging curve of the DSC for water. The phase-change peak temperature was the solidification temperature (*T_cp_*) and melting temperature (*T_dp_*) in the charging and discharging processes, respectively. The *T_cp_* during the charging process is higher than the starting temperature (*T_cs_*), which means that subcooling (or supercooled) occurs. The difference between *T_dp_* and *T_cp_* was defined as the *SD* of the sample [19,33,34]. The area of the charging and discharging peaks was calculated using DSC test software (Universal Analysis 2000, TA, New Castle, DE, USA) to obtain the phase-change heat for solidification (Δ*H_s_*) and for melting (Δ*H_m_*). For each test sample, the aforementioned fundamental characteristic and phase-change experiments were conducted five times. The obtained data were then averaged to obtain the final test results for all samples.

The uncertainty range of *k*, *μ*, *ρ*, *θ*, and the DSC refers to deviations from the relevant measuring instruments and temperature controller. The maximum uncertainty ranges of *k*, *μ*, *ρ*, *θ*, and the DSC were ±5.39%, ±2.24%, ±2.00%, ±0.29% (±0.1°), and ±1.47%, respectively. The experimental results are presented as a change ratio (*R*) to show the differences between the experimental results of water (*D_w_*) and those of the other samples (*D_s_*); *R* can be expressed as

*R* = [(*D_s_* − *D_w_*)/*D_w_*] × 100% (1)

## 4. Results and Discussion

Table 2 presents the test results and the change ratios of *k*, *μ*, *ρ*, and *θ* of the samples. Because the concentrations of SDBS, GP, and MNCBMs were low, no significant differences were observed in *k*, *μ*, and *ρ* among the samples. However, the *θ* of each sample differed greatly from that of water. SDBS addition to water markedly reduced the *θ* compared with no SDBS addition. Moreover, GP and MNCBM addition to the SDBS aqueous solution (base fluid) further reduced the *θ*. SDBS is a surfactant and can improve surface wettability and reduce the *θ* of a sample. The experimental results revealed that GP and MNCBM addition to the SDBS aqueous solution improved surface wettability of the test substrate and further reduced the *θ*. According to the theory of heterogeneous nucleation, a low *θ* contributes to such nucleation [7,9]; therefore, SDBS, GP, and MNCBM addition to water should enhance its ice nucleation efficiency.

**Figure 7:**
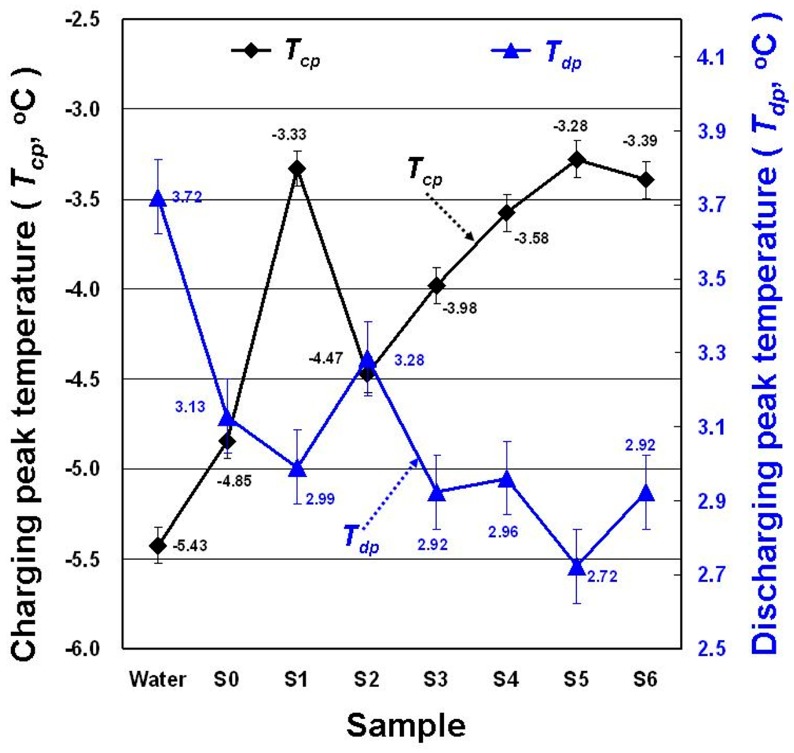
Changes in solidification temperature (*T_cp_*) and melting temperature (*T_dp_*) for the samples.

Figure 7 shows the *T_cp_* and *T_dp_* of each sample in the DSC experiment. *T_cp_* and *T_dp_* respectively represent the solidification (freezing) and melting temperatures in the DSC experiment. SDBS addition to water increased the *T_cp_* of water, and GP and MNCBM addition to the SDBS aqueous solution further enhanced the *T_cp_* of water. Compared with water, the *T_cp_* of the S5 showed the largest increase (2.15 °C). SDBS, GP, and MNCBM addition to water reduced its *T_dp_*. Compared with water, the *T_dp_* of the S5 exhibited the largest decline (1 °C). A PCM has higher *T_cp_*, which enables the charging process to occur at higher temperatures. Thus, the larger temperature difference between the PCM and the coolant of the chiller can be used for solidification phase-change to enhance heat transfer and shorten ice-storage time, thereby increasing charging process efficiency. The PCM with lower *T_dp_* had a larger temperature difference between PCM and air-conditioning load, and this enhanced heat-transfer efficiency in response to large air-conditioning load changes.

**Figure 8:**
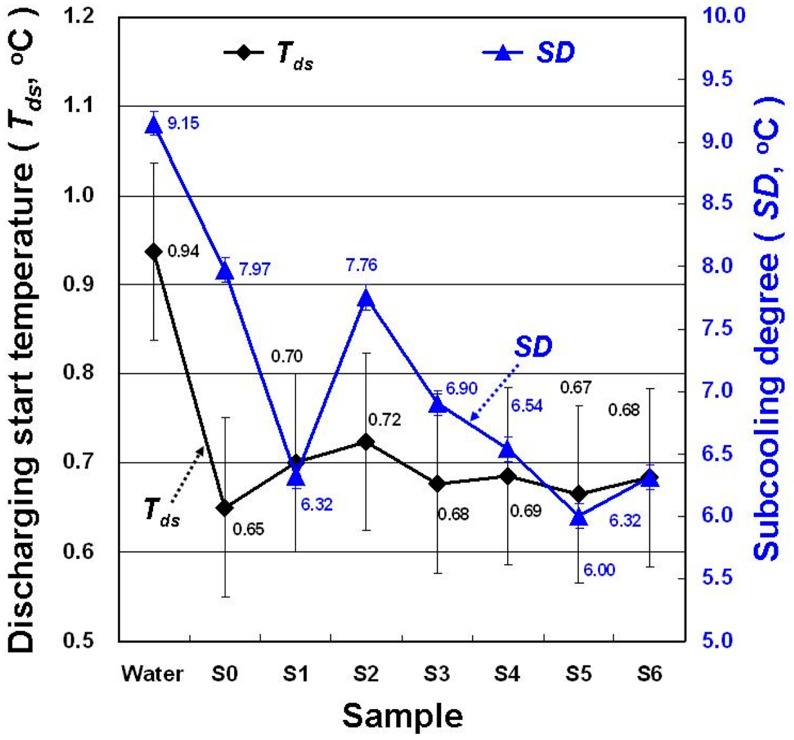
Changes in *T_ds_* and subcooling degree (*SD)* for the samples.

Figure 8 shows the discharging process start temperature (*T_ds_*) and *SD* of each sample in the DSC experiment. SDBS, GP, and MNCBM addition to water reduced the *T_ds_* and *SD* of water. Compared with water, the *T_ds_* and *SD* of the S0 and S5 exhibited maximum decline (0.29 °C and 34.40%, respectively). The lower *T_ds_* of the PCM indicated that the phase-change heat could be used for the air-conditioning load at lower temperatures. The PCM had a lower *SD*; thus, it could increase the evaporation temperature setting of the chiller to enhance its operating efficiency; SDBS, GP, or MNCBMs served as nucleating agents in water to reduce the *SD* of water.

**Figure 9:**
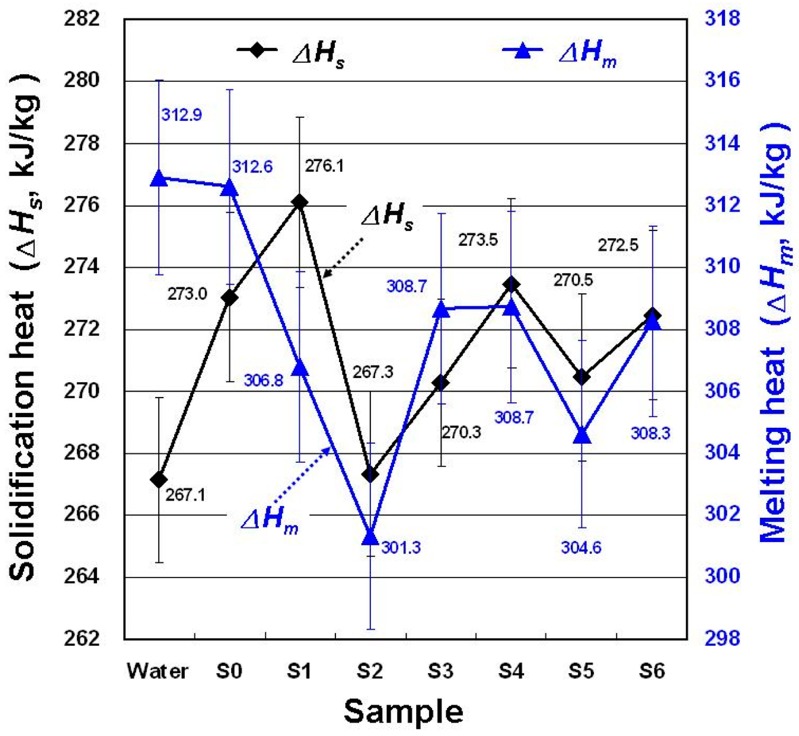
Changes in heat for solidification (*ΔH_s_*) and melting (*ΔH_m_*) for the samples.

Figure 9 displays the Δ*H_s_* and Δ*H_m_* of each sample in the DSC experiment. In theory, GP and MNCBM addition would not change the phase during ice nucleation or contribute to the phase-change heat [1]. Therefore, GP or MNCBM addition to water should reduce the phase-change heat of water. However, SDBS, GP, and MNCBM addition to water actually slightly increased the Δ*H_s_* of water. Adding nucleating agents such as SDBS, GP, or MNCBMs facilitated ice nucleation and resulted in higher Δ*H_s_* than that of water. When Δ*H_s_* is lower than Δ*H_m_*, Δ*H_s_* is mainly affected by the sample’s degree of crystallization, which is mainly affected by the DSC cooling rates. The phase-change heat differences among all samples were nonsignificant in this study, and compared with water, the change ratios of Δ*H_s_* and Δ*H_m_* in all samples were 0.07–3.36% and –0.10% to –3.70%, respectively. Therefore, in this study, no sample significantly enhanced the thermal storage density or reduced the volume of thermal storage tanks at the same thermal storage capacity.

The main factors affecting icing heterogeneity nucleation include the morphology and specific surface area of the added material, the interface characteristics between the added material and base fluid, the *k* of the sample, the dynamic viscosity (*ν* = *μ*/*ρ*) of the sample, and the *θ* of the sample [5,13,14,17,18,19,30]. The morphology of the added GP and MNCBMs was irregular and the interface characteristics between the added materials and base fluid were not further confirmed. Therefore, the influence of the morphology and interface characteristics of the added materials on the icing nucleation-related characteristics were not confirmed. In addition, the experimental data do not show either a high *k* or a low *ν* of sample can achieve a lower *SD*. This phenomenon should be attributed to the following two reasons: the addition of SDBS, GP, and MNCBMs to the water increases the hydrophilicity of water (reducing the *θ*); the GP and MNCBMs as nucleating agents affect the *SD* on each sample much higher than the *k* and *ν*. Therefore, the main factor for S5 with the lowest *T_dp_*, *SD*, and the highest *T_cp_* in this study should be because S5 has the smallest *θ*. 

Generally, for each 1 °C increase in the chiller evaporation temperature, chiller power consumption decreases by 2–3% [18]. Regarding variations in temperature, the *SD* and phase-change heat of each sample should serve as the key index. The *SD* of S5 was 34.40% (i.e., 3.15 °C) lower than that of water; therefore, S5 was the most promising material for use as a PCM in this study. The related literature shows that the maximum reduction ratio for *SD* of the water-based PCM added graphene oxide [3], carbon nanotubes [15,17,18], and graphene [14,15] is about 12–100%. In addition, the maximum reduction ratio of *SD* using copper [5], aluminum oxide [10,11], titanium dioxide [12,13,14], and silicon dioxide [14] as additives is about 12–78%. The sample (S5) of the maximum reduction ratio of *SD* in this study was not particularly excellent compared with the literature although it was within the range of reduction ratio. The reason is due to the excessive particle size of the current MNCBM suspended in CBSs and the lower *k* of CBSs. In the future, we can change the ball-milling time and mode of MNCBMs to reduce the particle size of MNCBMs and increase the specific surface area of MNCBMs to increase the *k* of CBS and reduce the *μ* of CBS, which may further reduce the *SD* of CBS during icing and improve cold storage efficiency.

## 5. Conclusions

In this study, MNCBMs were produced using the HPCM in an isoperibol oxygen bomb calorimeter with varying oxygen pressures; these MNCBMs were subsequently added to water to form CBSs as test samples for fundamental characteristic and phase-change experiments. The experimental results revealed that the material of the MNCBMs was mainly the same crystalline graphite as the original GP when the oxygen pressure was lower than 1.5 MPa. However, when oxygen pressure exceeded 1.5 MPa, the material mostly comprised crystalline graphite, amorphous graphite, and a small amount of defective diamond. No significant differences were observed in *k*, *μ*, and *ρ* among the samples because of low concentrations of SDBS, GP, and MNCBMs. SDBS, GP, and MNCBM addition to water improved the surface wettability of the test substrate and further reduced the *θ*. SDBS, GP, or MNCBM addition to water decreased the *SD* of water. The main factor for the CBS of S5 had the lowest *T_dp_*, *SD*, and the highest *T_cp_* in the DSC experiment should be because S5 has the smallest *θ*. Therefore, S5 was the most suitable PCM for all samples in this study and could contribute considerably to operation efficiency, energy saving, and carbon reduction in ISACSs.

## Figures and Tables

**Table 1:** Configuration of the experimental samples.

Composition	Sample No.							
	Water	BF	GP	0.5 MPa	1.0 MPa	1.5 MPa	2.0 MPa	2.5 MPa
		S0	S1	S2	S3	S4	S5	S6
GP (wt.%)	—	—	0.25	—				
MNCBMs (wt.%)	—	—	—	0.25				
Sodium dodecyl benzene sulfonate (SDBS) (wt.%)	—	0.4						
Defoamer (DF) (wt.%)	—	0.14						

**Table 2:** Results for fundamental characteristics.

Item		Sample NO.							
		Water	S0	S1	S2	S3	S4	S5	S6
Experimental data	*k* (W/(m·°C))	0.606	0.593	0.595	0.595	0.590	0.587	0.588	0.606
	*μ* (mPa·s)	0.80	0.80	0.80	0.80	0.80	0.80	0.85	0.82
	*ρ* (kg/m^3^)	997.05	997.70	999.23	999.30	999.07	999.30	999.33	999.13
	*θ* (deg.)	81.27	39.24	37.59	35.71	35.29	35.04	34.93	35.31
*R* (%)	*R*_k_	—	−2.15	−1.75	−1.90	−2.67	−3.15	−3.03	−0.08
	*R*_μ_	—	0.00	0.00	0.00	0.00	0.00	6.25	2.08
	*R*_ρ_	—	0.07	0.22	0.23	0.20	0.23	0.23	0.21
	*R*_θ_	—	−51.72	−53.75	−56.07	−56.58	−56.89	−57.03	−56.55
